# Exploring Demographics and Health as Predictors of Risk-Taking in UK Help-Seeking Veterans

**DOI:** 10.3390/healthcare6020058

**Published:** 2018-06-05

**Authors:** Rachel Ashwick, Shabeer Syed, Dominic Murphy

**Affiliations:** Combat Stress, Tyrwhitt House, Leatherhead, Surrey KT22 0BX, UK; rachel.ashwick@btconnect.com (R.A.); shabeer.syed@combatstress.org.uk (S.S.)

**Keywords:** veterans, posttraumatic stress disorder, TBI, risk, alcohol, fighting, driving, mental health

## Abstract

Risk-taking amongst veterans has severe consequences, yet few studies have examined factors that may predict risk-taking in help-seeking veteran populations. This paper presents a cross-sectional study involving a random sample of 667 UK help-seeking veterans, investigating the role of demographics, mental health and physical health presentations on the propensity for risk-taking. Out of 403 (73.4%) veterans, 350 (86.8%) reported risk-taking in the past month. We found that younger age, being in a relationship, probable PTSD, common mental health difficulties and traumatic brain injury were significantly associated with risk-taking. Additionally, a direct association was found between increased risk-taking and PTSD symptom clusters, including higher hyperarousal, elevated negative alterations in mood and cognition. Our findings provide initial evidence for demographic and mental health presentations as predictors of risk-taking in help-seeking veterans. Further research and longitudinal studies are needed to facilitate valid risk assessments, and early intervention for veteran services.

## 1. Introduction

Nowadays, risk-taking in military populations is often a substantial public health concern and a leading cause of preventable deaths among military personnel [1]. Reports estimate that 31% of these deaths are caused by accidents linked to risk-taking post-deployment [2]. This data mirrors studies demonstrating that veterans involved in lethal road traffic accidents were more likely to drive above the speed limit, not wear a seatbelt, smoke and misuse alcohol [3]. Violence and fighting have also been highlighted as a major concern in military populations, with prevalence estimates ranging from 13% to 58% [4]. The impact of risk-taking is further compounded by the significant associated health care costs, with alcohol and smoking attributed to cost the US military over $1.2 annually [5]. 

Despite consistent reports on the impact of risk-taking among military personnel, few studies have investigated help-seeking populations. Help-seeking veterans are outlined as a specific high-risk group for risk-taking due to their elevated health difficulties [6]. A longitudinal study found that help seekers who suffer from physical and mental health problems often engage in various risk-taking behavior long after leaving the military [7], further exacerbating their difficulties [8]. Worryingly, reports illustrate that 62.4% of veteran deaths following treatment were linked to behaviors that could be prevented [1]. 

Strong theoretical frameworks for elevated risk-taking in help-seeking veterans, however, remain ambiguous. By nature, military personnel are exposed to situations of uncertainty and violence, with shooting, fighting, and inter-unit competitions forming core components of basic training [9]. Some frameworks illustrate that the normalization of these behaviors is linked to automatic behaviors in civilian life [10]. Moreover, the elevated prevalence of mental health difficulties among help-seekers, including traumatic brain injuries and Post Traumatic Stress Disorder (PTSD), have been associated with biological changes in the brain, affecting attention and decision making [11,12,13,14]. Research conducted on US veterans shows that PTSD symptoms consistently correlate to increased substance misuse, suicide attempts, thrill-seeking and aggression [15]. Other theories propose that individuals who join the military are naturally more inclined to risk-taking [16]. A report published by the Department of Defence on 28,546 serving personnel highlighted that 78% classified as sensation-seekers [17]. 

Whilst these initial findings provide relevance to inform prevention for US personnel, corresponding research into risk-taking among UK veterans remains scarce. In comparison to the US, UK personnel often differ in terms of military service, socio-demographics, and health difficulties, and the availability of post-deployment care shadows the US [18]. Insights into the predictors of risk-taking among UK help-seeking veterans are urgently needed to advance risk assessments and interventions across healthcare providers. This is the first study to examine the potential predictors of risk-taking behavior in a nationally representative sample of UK help-seeking veterans.

## 2. Materials and Methods 

### 2.1. Ethics

This study received approval from the Combat Stress Ethics Committee. Participants gave informed consent and all data were anonymized to ensure no identifiable participant information was reported. 

### 2.2. Design

The current study employed a cross-sectional design to explore the predictors of risk-taking in help-seeking veterans. The primary outcome variable was a composite variable of risk-taking, aligning with previous epidemiological methods [19]. Potential predictors included demographics and health presentations.

### 2.3. Participants

Participants were required to be UK veterans, defined as having served a minimum of one day in the UK military and have contacted Combat Stress (CS) between January 2015 and January 2016. CS is a national UK charity providing clinical mental health services for veterans with PTSD and is commissioned by the National Health Service. To create a representative sample of UK help-seeking veterans, no exclusion criteria were applied. The total number of veterans meeting our criteria was 3335. From this, a 20% (*n* = 667) randomly selected sample was obtained via random number generation. 403 participants completed the study and 51 opted out. After removing any participants who were deceased (*n* = 4) or had invalid contact details (*n* = 63), the final adjusted response rate was 67.2% (*n* = 403). 

### 2.4. Measures

#### 2.4.1. Demographics.

A self-report questionnaire collected demographics, including; sex (male/female), age, relationship status (single/in a relationship), employment status (employed/unemployed), whether the participant had left the Armed Forces early (<5 years; yes/no) and the length of time taken to contact Combat Stress (≥5 years; yes/no). Age was grouped into four categories: less than 35, 35 to 44, 45 to 54 and 55 years and over to aid clinicians in identifying the age bracket most at risk. 

#### 2.4.2. Health Presentations.

PTSD was assessed using the Posttraumatic Checklist for the DSM-5 (PCL-5; [20]), which has shown high validity (sensitivity 0.88; specificity 0.69) and reliability in treatment-seeking veterans [21]. The PCL-5 comprises of 20 items with a Likert scale ranging from 0 (not at all) to 4 (extremely) asking how much in the past month individuals have been affected by symptoms of PTSD. A total score was calculated and participants who scored 38 or more met criteria for probable PTSD [20]. Individual scores for the symptom clusters were summed to investigate the association between intrusive thoughts (items 1 to 5), avoidance (items 6 to 7), negative alterations (questions 8 to 14), hyperarousal (items 15 to 20) and risk-taking. 

Common mental health difficulties were assessed using the General Health Questionnaire (GHQ-12) due to the high validity (sensitivity 0.76; specificity 0.83) in clinical populations [22]. The GHQ-12 uses 12 items with a Likert scale ranging from 0 to 3. The last six item scores were reverse scored to match the same scale as the first six. A score above four marked a probable diagnosis of anxiety and depression [23]. 

Traumatic Brain Injury (TBI) was assessed using the Brain Injury Screening Index (BISI; [24]). The BISI has been used in previous veteran studies [8]. Participants reported their most serious head injury and endorsement of any one symptom indicated a TBI. 

Finally, physical health problems were measured using the self-reported question “do you have any problems relating to the following physical health areas? Tick all that apply”. A list of 13 problems was taken from an NHS screening tool used within a South East Hospital in England. These included; diabetes, cardiovascular problems, blood pressure, respiratory problems, liver or kidney problems, amputation, neurological problems, digestive problems, chronic pain, poor mobility, hearing impairment, sight impairment, communication problems. The total number of problems was summed and scores were then evenly split into either a ‘high-risk’ or ‘low-risk’ group for physical health problems, as informed by the sample median post-hoc [25]. 

#### 2.4.3. Risk-Taking Behaviors

The primary outcome ‘risk-taking behavior’ was a composite variable based on previously established military risk-taking behaviors, including; heavy smoking [26], alcohol misuse [27], risky driving [28], and fighting [3]. Cases of risk-taking behaviors across variables were summed to create a continuous mean risk score [19]. 

Survey items regarding smoking asked participants whether or not they smoked and if so, how many cigarettes they smoked a day. If participants smoked ≥20 cigarettes a day, they were classed as risky smokers [28].

Risky alcohol consumption was measured using the Alcohol Use Disorders Identification Test (AUDIT) to identify high drinking levels due to the high reliability and validity (sensitivity 0.86; specificity 0.89) showed in clinical and military populations [29,30]. The AUDIT comprises of 10 items rating quantity and frequency of alcohol consumption over the past month on a scale of 0 to 4, where 0 represented ‘never’ and 4 was ‘daily or almost daily’. A score of 16 or above was classed as risky drinking [31].

Risky driving was assessed using a measure adapted from a King’s College study on risk-taking in military personnel [28]. Participants were asked the following questions: how fast they drive in a built-up area using forced-choice options: (1) within 5 mph of the limit (2) 6–10 mph above the limit and (3) more than 10 mph over. Participants were then asked how fast they drive on the motorway: (1) within 10 mph of the limit (2) 11–20 mph above the limit and (3) more than 20 mph over. If participants scored in the highest category in either question (3 points), this indicated risky driving [28].

Fighting was examined through the Walter Reed Aggression Scale (WRA-4). This measure was chosen due to it being specifically designed for military populations and used in a range of previous veteran studies [3,32,33]. The measure comprised of four items rating both verbally and physically aggressive behavior on a 5-point Likert scale, where 1 represented ‘never’ and 5 represented ‘5 or more times’. Veterans were asked how often they exhibited the fighting behaviors in the past month: “got angry at someone and yelled or shouted at them,” “got angry with someone and kicked or smashed something, slammed the door, punched the wall”, “got into a fight with someone and hit the person,” or “threatened someone with physical violence.” Answering ‘yes’ to any of these behaviors within the past month indicated fighting behavior [33].

### 2.5. Procedure

The data collection for this study was conducted at Combat Stress in Leatherhead from January to September 2016. The questionnaire was first mailed to participants three times and then remaining non-responders were phoned by the researcher to minimize response and selection bias. After three calls, no additional contact attempts were made. 

### 2.6. Statistical Analysis

The data was cleaned using frequency lists to check for invalid characters, numbers outside of the valid range and missing values, to minimize human errors. A multiple imputation protocol was put in place where a mean score would only be calculated to replace a missing value when less than 20% of the questionnaire data were missing; otherwise, data were excluded [34].

A risk variable was created from the total scores of each measure (smoking, alcohol misuse, risky driving, and fighting) which were coded according to their clinical cutoff scores. Scores above the clinical cut-off point were coded as ‘high-risk’ as they met criteria for caseness indicating risky behavior, and scores below the cut-off were coded as ‘low-risk’. Once binary variables for each variable were created, the risk-taking variables were combined to create a continuous composite primary outcome variable of ‘risk-taking behavior’ as guided by the additive model by Pickett et al. (2002) [19].

First, unadjusted linear regressions were employed to explore the relationship between each demographic variables (age, sex, relationship status, employment status, early service leaver status and time to contact CS) and the composite risk-taking behavior. The same analyses were repeated adjusting for these demographics.

Second, separate linear regression models were fitted to each health outcome (PTSD, common mental health difficulties, TBI and physical problems) to explore predictors of risk-taking. A multivariate regression was conducted adjusting for each of the other health outcomes. For example, if PTSD was examined, common mental health difficulties, TBI, and physical health were adjusted for. In addition, all analyses adjusted for age and relationship status.

Finally, a linear regression model was independently fitted to each PTSD symptom cluster, with risk-taking as the primary outcome. To minimize the influence of common confounders, subsequent analyses adjusted for the other symptom clusters, age and relationship status. All analyses were conducted in 2017 using STATA Version 13.0 (StataCorp LLC; College Station, TX, USA).

## 3. Results

### 3.1. Sample Characteristics

Of the 403 participants completing the study, 386 were male (96%) with an average age of 50.9 years (SD = 12.6, range: 22–92). In total, 68% were in a relationship and 69% were employed. Only 11% of the sample had left the UK Armed Forces early and 48% had taken longer than 5 years to seek help from CS. In terms of health, 82% of the sample met criteria for probable PTSD, 73% for common mental health difficulties, 48% for TBI and 28% for high-risk physical health problems. No differences were found between responders and non-responders [35]. 

Table 1 presents the proportions of risk-taking behaviors. 350 (86.8%) of the participants engaged in any type of risk-taking in the past month. Specifically, 77.9% engaged in fighting, 40.2% in risky drinking, 15.4% in risky smoking, and 7.2% in risky driving. Moreover, 13.2% engaged in no risk-taking behavior, 44.9% in one, 30.5% in two, 10.9% in three and 0.5% in all four behaviors.

### 3.2. Predictors of Risk-Taking

Following adjustment for demographics, a negative relationship was found between those aged above 55 engaging in lower risk-taking relative to those under 35 years (adjusted coefficient: −0.70, 95% CI: −1.02 to −0.38, *p* < 0.0001). Similarly, a negative relationship was established in those aged 35 to 44 compared to those under 35 (adjusted coefficient: −0.32, 95% CI: −0.66 to −0.00, *p* = 0.049). In contrast, a positive association was found between participants who were in a relationship exhibiting greater risk-taking behaviors compared to those without a partner (adjusted coefficient: 0.46, 95% CI: 0.26 to 0.66, *p* < 0.0001). These findings are presented in Table 2. 

A positive relationship was found between those with PTSD and an increase in risk-taking behaviors (adjusted coefficient: 0.38, 95% CI: 0.13 to 0.63, *p* = 0.003). TBI was also found to be significantly associated with greater risk-taking compared to those without a TBI (adjusted coefficient: 0.22, 95% CI: 0.04 to 0.40, *p* = 0.017). Finally, a positive relationship was found between those with common mental health difficulties and greater risk-taking (adjusted coefficient: 0.24, 95% CI: 0.02 to 0.46, *p* = 0.032). No association was found between physical health problems and risk-taking (Table 3).

Table 4 presents the associations between symptom clusters of PTSD and risk-taking. A positive relationship was found between hyperarousal and risk-taking (adjusted coefficient: 0.05, 95% CI: 0.02 to 0.07, *p* = 0.001), with a small increase in risky behaviors per each increase of one in the score. Additionally, moderate evidence for a positive relationship between negative alterations and risk-taking were found (adjusted coefficient: 0.02, 95% CI: 0.00 to 0.05, *p* = 0.022). 

## 4. Discussion

### 4.1. Main Findings

This was the first study to explore demographics and health as predictors of risk-taking in UK help-seeking veterans. Veterans who were younger, in a relationship, met criteria for probable PTSD, common mental health difficulties, and TBI were more likely to engage in risk-taking behaviors. For PTSD symptom clusters, higher hyperarousal and negative alterations in mood and cognition were significant predictors of risk-taking.

#### 4.1.1. Risk-Taking Behaviors

The majority of help-seeking veterans engaged in risk-taking behaviors, of which fighting was the most common, followed by heavy drinking, smoking and, risky driving. These findings support the theory that veterans tend to use alcohol and smoking as emotional numbing strategies for health difficulties [36]. As high levels of drinking have been associated with a range of physical, social and psychological problems as well as a reduced quality of life, the high rate in help-seeking veterans indicates the need for interventions to reduce this behavior [37]. Additionally, fighting was found to be six times higher in help-seeking veterans compared to previous findings of military personnel [38]. Given the high proportion of PTSD within this population group, this high fighting prevalence may be due to neurobiological changes in the brain following trauma and difficulties with emotion regulation [38,39].

Finally, a previous study found that individuals who engaged in one type of risk-taking behavior were more likely to engage in others [40], whereas our results found that the majority of help-seeking veterans exhibited only one behavior. This lower number of risk-taking may be due to the impact of social normalization, whereby, once they have left the military there is less pressure from their peers to adhere to social expectations [41]. 

#### 4.1.2. Demographics and Risk-Taking

Veterans aged 35–44 and over the age of 55 were significantly less likely to engage in risk-taking compared to those under 35. Studies have suggested that aging leads to decreased levels of testosterone and neurotransmitters such as dopamine and serotonin [42]. As these chemicals are linked to impulsivity, this observation supports the idea that age reduces impulsivity and thus, risk propensity. Nevertheless, no significant correlation was found between risk-taking and veterans aged 45–54 compared to those under 35. 

While previous studies have determined that males have a greater biological propensity for risk-taking due to higher testosterone [43], no sex differences in risk-taking were found. However, this may have been due to the low number of females in the sample limiting the power to detect differences. Another divergent finding was that veterans who were in a relationship were more likely to take risks than single veterans [44]. The differences in our findings may be due to the impact that trauma and mental health difficulties have on relationship quality [45], which can then lead to emotional regulation difficulties and risk-taking [46].

#### 4.1.3. Health and Risk-Taking

Veterans who met criteria for common mental health problems, TBI and PTSD were more likely to engage in risk-taking. Aligning with the emotional regulation theory in compassion-focused therapy [47], trauma disrupts cognitive functioning resulting in overactive threat and drive systems and underactive soothing systems [48]. As a result, veterans may have higher perceptions of threat and respond to this via aggressive behavior. Additionally, underactive soothing systems may lead to difficulty experiencing positive emotions and veterans may seek positive reinforcement via behaviors that increase their adrenaline levels [49]. 

Conversely, no differences were found in risk-taking between high and low physical health groups. Ben-Zur and Zeidner (2009) proposed that physical health difficulties resulting from trauma may reduce an individual’s attention span [50]. An individual may become more focused on their physical recovery, that they neglect other aspects of their life and take more risks. As the physical health questionnaire did not account for the severity of physical health problems, this may explain the lack of differences between the high and low groups, as an individual who has one severe medical problem may be equally as affected by their difficulty as someone who has numerous problems. Conversely, the presence of any physical health problem may impair the individual’s ability to engage in risky behavior.

Finally, findings illustrated a significant dose-response effect between risk-taking and PTSD symptoms of hyperarousal and negative alterations of mood. Previous examinations of symptom clusters and risk propensity have provided mixed associations. However, these findings parallel that of a US study showing hyperarousal to be linked to aggression in treatment-seeking veterans and can be explained in terms of increased dopamine and serotonin impairing decision-making [51]. Conversely, individuals who experience more severe negative alterations in mood and cognitions result in a lower regard for negative outcomes and difficulty experiencing positive emotions [52].

### 4.2. Strengths and Limitations

Given the high response rate and homogeneity between responders and non-responders [35], this study benefits from good external reliability and generalizability. However, recruiting from one charity specializing in PTSD may have affected the representativeness of the data, as participants were likely to have more acute problems or mainly PTSD. Nevertheless, randomization and recruiting from a national charity increased representativeness. As the sample comprised of primarily males (96%) findings may not be representative of female help-seekers. However, as 88.9% of UK veterans are male [53], this reflects the overall low number of females.

While the current study measures (PCL-5, GHQ-12, BISI, AUDIT) are widely used in clinical practice, a clinical interview with a trained professional is required to establish an accurate mental health diagnosis. As such, the current case criteria only provide an indicative screening that the individual may have the mental health difficulty. Moreover, the use of screening tools may have introduced bias or over-reported symptoms [54]. 

A further limitation was the use of the WRA-4 to assess fighting. The scoring of the WRA-4 does not distinguish between verbal and physical violence which may have led to an overestimation of physically violent behavior. Nevertheless, verbal aggression can have a profound effect on the psychological well-being of the victim, so must be acknowledged as a form of violence [55].

Finally, the dichotomization of variables could have led to the loss of rich data, increasing the risk of type two errors [56]. Variables were dichotomized due to the similarity of the answer categories. For example, ‘divorced’ and ‘single’ naturally fell into the same category of ‘not in a relationship’. In addition, in some answer categories, there were insufficient responses endorsing an option to produce reliable results with continuous variables. 

### 4.3. Implications 

The findings of this study have a range of clinical implications. As several predictors were found for risk-taking, it is important these individuals are identified early-on through additional risk assessments on risk-taking behaviors, are prioritized for treatment and continually monitored upon discharge. Specifically, veterans who are younger, in a relationship, screen positive for probable PTSD, common mental health difficulties and TBI should undertake an additional risk assessment regarding risk-taking behaviors. Currently, standard protocols target suicidal behaviors and additional assessments are needed for other behaviors such as alcohol misuse, smoking, risky driving and fighting. Specific psycho-education sessions could be provided focusing on the consequences of risky behavior, the biological and psychological mechanisms, identifying triggers and developing alternative coping strategies which may increase awareness and reduce these behaviors amongst veterans [57].

Future longitudinal research is required to examine how factors such as demographics and health presentations affect veteran risk-taking over time as well as examining military characteristics. As interventions targeting risk-taking are currently limited, clinical research is needed to develop an evidence-based intervention to reduce risk-taking in help-seeking veterans. One pilot study in the US has begun to investigate the effect of a group-based Cognitive Behavioral Therapy intervention on risky driving but is limited to a small sample size, no control group and a short one-month follow-up [57]. Future research is needed in the UK to design an intervention that accounts for multiple risk-taking behaviors in help-seeking veterans. The implications of a robust and focused research program could be far-reaching, offering important benefits for help-seeking veterans worldwide. 

## 5. Conclusions

This was the first study to explore demographics and health as predictors of risk-taking in a sample of UK help-seeking veterans. 87% of our sample had engaged in risk-taking in the past month, with fighting (77.9%) and heavy drinking (40.2%) most common. Veterans who were younger and in a relationship were more likely to engage in greater risk-taking, alongside those who met case criteria for probable common mental health difficulties, PTSD and TBI. Finally, a direct association was found between higher hyperarousal, more negative alterations in mood and cognition and increased risk-taking. The development of additional screening and focused interventions for risk-taking is vital to lessen morbidity rates and preventable deaths among veterans.

## Figures and Tables

**Table 1:** The proportion of veterans engaging in each category of risk-taking.

Risk-Taking Behavior	*n*	Percentage (%)
**Type of behavior**		
Heavy drinking	162	40.2
Smoking	62	15.4
Fighting	314	77.9
Driving	29	7.2
Any	350	86.8
**Number of behaviors**		
0	53	13.2
1	181	44.9
2	123	30.5
3	44	10.9
4	2	0.5

**Table 2:** Association between demographics and risk-taking behavior.

Predictor	*n* (%)	Mean Risk Score (M)	Unadjusted Coefficient (95% CI)	*p* Value	Adjusted Coefficient ^a^ (95% CI)	*p* Value
**Sex**						
Male	386 (96)	0.86	1.0	-	1.0	-
Female	17 (4)	0.65	−0.21 (−0.69, 0.27)	0.389	−0.34 (−0.81, 0.12)	0.150
**Age**						
<35	49 (12)	1.27	1.0	-	1.0	-
35–44	95 (24)	0.92	−0.35 (−0.68, −0.02)	0.040 *	−0.33 (−0.66, −0.00)	0.049 *
45–54	110 (27)	0.93	−0.34 (−0.66, −0.01)	0.042 *	−0.32 (−0.65, 0.00)	0.051
55+	149 (37)	0.61	−0.65 (−0.97, −0.34)	<0.0001 ***	−0.70 (−1.02, −0.38)	<0.0001 ***
**Relationship**						
Single	130 (32)	1.17	1.0	-	1.0	-
In a relationship	273 (68)	0.70	0.47 (0.27, 0.67)	<0.0001 ***	0.46 (0.26, 0.66)	<0.0001 ***
**Employment**						
Working	126 (31)	0.80	1.0	-	1.0	-
Not working	277 (69)	0.87	0.07 (−0.14, 0.28)	0.518	0.14 (−0.07, 0.35)	0.190
**Early service leaver**						
No	357 (89)	0.83	1.0	-	1.0	-
Yes	46 (11)	1.00	0.17 (−0.13, 0.47)	0.269	0.04 (−0.26, 0.34)	0.786
**Time to contact**						
<5 years	210 (52)	0.85	1.0	-	1.0	-
>5 years	193 (48)	0.84	−0.01 (−0.20, 0.19)	0.937	0.14 (−0.06, 0.33)	0.180

* *p* < 0.05, *** *p* < 0.001. There are no missing values. ^a^ Adjusted for sex, age, relationship status, employment status, early service leaver and time to contact services.

**Table 3:** Association between health outcomes and risk-taking behavior.

Predictor	*n* (%)	Mean Risk Score (M)	Unadjusted Coefficient (95% CI)	*p* Value	Adjusted Coefficient ^a^ (95% CI)	*p* Value
**PCL-5**						
Score < 38	72 (18)	0.40	1.0	-	1.0	-
Score => 38	331 (82)	0.95	0.54 (0.30, 0.79)	<0.0001 ***	0.38 (0.13, 0.63)	0.003 **
**GHQ-12**						
Score < 4	110 (28)	0.57	1.0	-	1.0	-
Score => 4	290 (73)	0.96	0.38 (0.17, 0.60)	<0.001 ***	0.24 (0.02, 0.46)	0.032 *
**TBI**						
No	211 (52)	0.73	1.0	-	1.0	-
Yes	192 (48)	0.98	0.26 (0.07, 0.45)	0.008 **	0.22 (0.04, 0.40)	0.017 *
**Physical problems**						
Low group	289 (72)	0.88	1.0	-	1.0	-
High group	114 (28)	0.77	−0.11 (−0.32, 0.11)	0.327	−0.01 (−0.22, 0.21)	0.936

* *p* < 0.05, ** *p* < 0.01, *** *p* < 0.001. There are 3 (0.7%) missing values for GHQ-12 score; ^a^ Adjusted for PCL-5, GHQ-12, TBI, physical problems, age and relationship status.

**Table 4:** Association between PTSD symptom clusters and risk-taking behavior.

Predictor	Unadjusted Coefficient (95% CI)	*p* Value	Adjusted Coefficient ^a^ (95% CI)	*p* Value
**Intrusions**				
No	1.0	-	1.0	-
Yes	0.04 (0.02, 0.06)	<0.0001 ***	−0.01 (−0.03, 0.02)	0.644
**Avoidance**				
No	1.0	-	1.0	-
Yes	0.07 (0.03, 0.11)	<0.001 ***	−0.04 (−0.09, 0.01)	0.130
**Negative alterations**				
No	1.0	-	1.0	-
Yes	0.05 (0.03, 0.06)	<0.0001 ***	0.02 (0.00, 0.05)	0.022 *
**Hyperarousal**				
No	1.0	-	1.0	-
Yes	0.06 (0.05, 0.08)	<0.0001 ***	0.05 (0.02,0.07)	0.001 ***

* *p* < 0.05, *** *p* < 0.001, ^a^ Adjusted for intrusions, avoidance, negative alterations, hyperarousal, age and relationship status.
